# A comparative study of two different approaches for the incorporation of silver nanoparticles into layer-by-layer films

**DOI:** 10.1186/1556-276X-9-301

**Published:** 2014-06-13

**Authors:** Pedro Jose Rivero, Javier Goicoechea, Ignacio Raul Matias, Francisco Javier Arregui

**Affiliations:** 1 Nanostructured Optical Devices Laboratory, Electric and Electronic Engineering Department, Public University of Navarra, Edif. Los Tejos, Campus Arrosadía, Pamplona 31006, Spain

**Keywords:** Silver nanoparticles, Thin films, *In situ* synthesis process, Layer-by-layer embedding deposition technique

## Abstract

In this work, a comparative study about the incorporation of silver nanoparticles (AgNPs) into thin films is presented using two alternative methods, the *in situ* synthesis process and the layer-by-layer embedding deposition technique. The influence of several parameters such as color of the films, thickness evolution, thermal post-treatment, or distribution of the AgNPs along the coatings has been studied. Thermal post-treatment was used to induce the formation of hydrogel-like AgNPs-loaded thin films. Cross-sectional transmission electron microscopy micrographs, atomic force microscopy images, and UV-vis spectra reveal significant differences in the size and distribution of the AgNPs into the films as well as the maximal absorbance and wavelength position of the localized surface plasmon resonance absorption bands before and after thermal post-treatment. This work contributes for a better understanding of these two approaches for the incorporation of AgNPs into thin films using wet chemistry.

## Background

The development of nanostructured advanced materials based on the incorporation of metal nanoparticles has attracted the attention of the researchers [1-5]. The optical spectra of the metal nanostructures show an attractive plasmon resonance band, known as localized surface plasmon resonance (LSPR), which occurs when the conductive electrons in metal nanostructures collectively oscillate as a result of their interaction with the incident electromagnetic radiation [6,7]. Such nanoplasmonic properties of the metal nanostructures are being investigated because of their unique or improved antibacterial, catalytic, electronic, or photonics properties [8-15]. In addition, their excellent optical properties make them ideal to use in optical fiber sensors in detecting physical or chemical parameters [16,17].

A wide variety of methodologies are focused on the synthesis of metal nanoparticles with a fine control of the resultant morphology [18-24]. Of all them, chemical reduction methods from metal salts (i.e., AgNO_3_ or HAuCl_4_) are one of the most studied using adequate protective and reducing agents due to their simplicity [25-29]. Very recently, the high versatility of the poly(acrylic acid, sodium salt) (PAA) has been demonstrated as a protective agent of the silver nanoparticles because of the possibility of obtaining multicolor silver nanoparticles with a high stability in time by controlling the variable molar ratio concentration between protective and reducing agents [30]. This weak polyelectrolyte (PAA) presents carboxylate and carboxylic acid groups at a suitable pH, being of great interest for the synthesis of metal nanoparticles. Specifically, the carboxylate groups of the PAA can bind silver cations, forming positively charged complexes, and a further reduction of the complexes to silver nanoparticles takes place [31-33].

One approach for incorporating metallic nanoparticles into thin films is based on *in situ* chemical reduction of silver cations to zero valent nanoparticles into a previously fabricated host matrices used as a template. This *in situ* synthesis process of metallic nanoparticles can be applied to several well-known deposition techniques such as sol-gel process [34], electrospinning [35], or layer-by-layer (LbL) assembly [36]. Among of all them, LbL assembly shows a higher versatility for tailoring nanoparticles due to the use of polyelectrolytes with specific functional groups [37]. Furthermore, a thermal post-treatment of the films makes possible the fabrication of chemically stable hydrogels [35] because a covalent cross-link via amide bonds between the polymeric chains of the polyelectrolytes has been induced [38-40] with a considerable improvement of their mechanical stability.

In this work, two weak polyelectrolytes, poly(allylamine hydrochloride) (PAH) as a cationic polyelectrolyte and PAA as an anionic polyelectrolyte, have been chosen to build the multilayer structure. The pH-dependent behavior of the PAA makes possible to control the proportion of carboxylate and carboxylic acid groups [41-44]. The carboxylate groups are responsible of the electrostatic attraction with the positive groups of the PAH, forming ion pairs to build sequentially adsorbed multilayers in the LbL assembly. In addition, the carboxylic acid groups are known as nanoreactor host sites which are available for a subsequent metal ion exchange with the proton of the acid groups. More specifically, the carboxylic acid groups are responsible of binding silver cations via metal ion exchange (loading solution). Once silver ions have been immobilized in the films, a chemical reduction of the silver ions to silver nanoparticles (AgNPs) takes place when the films are immersed in the reducing solution. Several approaches have been presented in the bibliography using different loading and reduction agents as well as weak or strong polyelectrolytes [45-49]. Nevertheless, weak polyelectrolyte LbL templates (such as PAH and PAA) offer the additional advantage of an adjustable pH-dependent degree of ionization, which is a key parameter when *in situ* synthesis process (ISS) approach is used.

Alternatively, AgNPs-loaded LbL films can be built up using polyelectrolyte-capped metal nanoparticles. The use of PAA as a protective agent of the silver nanoparticles (PAA-AgNPs) plays a key role for a further incorporation into LbL films [30]. The carboxylate groups at a specific pH value are used to build the sequentially adsorbed multilayer structure with a cationic polyelectrolyte, preserving their aggregation of the AgNPs into the LbL films [50]. Henceforward, this approach of a successive incorporation of AgNPs of a specific morphology into LbL films will be referred as layer-by-layer embedding (LbL-E) deposition technique.

In this work, a comparative study about the synthesis and incorporation of AgNPs into thin films obtained by layer-by-layer assembly is presented using two alternative chemical methods. The first methodology is the ISS which is based on a first step of thin film fabrication, and then a second step where the synthesis of silver nanoparticles into the films is performed. The second methodology is the LbL-E deposition technique which follows a different order because firstly silver nanoparticles of a specific shape are synthesized, and then their incorporation into thin films using the LbL assembly is performed. Although both processes use the same reagents, remarkable differences related to the size, distribution, or maximal wavelength position of the LSPR band have been observed. Additionally, a thermal post-treatment was performed to fabricate stable hydrogel films with a better chemical stability via cross-link of the polymeric chains. This comparative study can be useful to the further design of advanced hybrid coatings based on metallic nanoparticles and polymeric materials.

## Methods

### Materials

Poly(allylamine hydrochloride) (Mw 56,000), poly(acrylic acid, sodium salt) 35 wt.% solution in water (PAA) (Mw 15,000), silver nitrate solution (> 99% titration, 0.1 N AgNO_3_), and dimethylamine borane complex (DMAB) were purchased from Sigma-Aldrich (St. Louis, MO, USA) and used without any further purification. Aqueous solutions of 0.01 M of both PAH and PAA were prepared using ultrapure deionized water (18.2 MΩ) and adjusted to pH values 7.0 and 9.0 by the addition of a few drops of HCl or NaOH 1 M.

### Fabrication of the thin films

All the thin films have been fabricated using a 3-axis Cartesian robot from Nadetech Innovations SL (Sarriguren, Spain). The LbL assembly was performed by sequentially exposing the glass slides to cationic and anionic polyelectrolytes with an immersion time of 2 min. A rinsing step in deionized water was performed between the two polyelectrolyte baths. The combination of a cationic monolayer with an anionic monolayer is called bilayer. More details of the LbL assembly can be found elsewhere [37].

### *In situ* synthesis of the silver nanoparticles

This process starts with a first step of a multilayer coating fabrication using the LbL assembly of cationic (PAH) and anionic (PAA) polyelectrolytes. A second step is where the ISS of the AgNPs into the polymeric coating was carried out.

The polymeric thin films are firstly immersed in an aqueous solution of silver nitrate (AgNO_3_ 0.01 N) at room temperature for 5 min, removed, and rinsed with ultrapure water. Then, once the silver ions have been incorporated into films via ion exchange, a further *in situ* chemical reduction of the silver cations (Ag^+^) to silver nanoparticles (Ag^0^) was performed at room temperature. The films are immersed in an aqueous solution of dimethylamine borane complex (DMAB 0.01 N) for 5 min, removed, and rinsed with ultrapure water.

### Layer-by-layer embedding deposition technique

This synthesis process is based on a first step of synthesis of silver nanoparticles with a desired shape, and then a second step where a further incorporation of the synthesized silver nanoparticles into a thin film using the LbL-E deposition technique is performed.

Silver nanoparticles have been synthesized at room temperature via chemical reduction process of an aqueous solution of silver precursor (AgNO_3_) with an aqueous solution of reducing agent (DMAB). More details of the synthesis can be found elsewhere [30]. In LbL-E, the PAA functionalized AgNPs were used as polyanion (PAA-AgNPs) in the LbL protocol, as it was described in ‘Fabrication of the thin films’ section.

### Thermal post-treatment

A thermal post-treatment was carried out in the resultant LbL films using temperatures from 50°C to 200°C in a furnace for a period of time of 2 h. The heat-treated cross-linked films have enhanced durability when immersed in aggressive conditions for several hours (buffer solution pH 10) and no delamination of the films was observed, while untreated films were severely damaged.

### Characterization of the thin films

UV-vis spectroscopy (UV-vis) was used to characterize the optical properties of the silver nanoparticles incorporated into the thin films. Measurements were carried out with a Jasco V-630 spectrophotometer (Jasco Inc., Easton, MD, USA).

Atomic force microscopy (AFM) and scanning electron microscopy (SEM) were used to characterize both the distribution of the nanoparticles and the morphology of the resultant thin films. The samples were scanned using a Veeco Innova AFM (Veeco Instruments, Inc., Plainview, NY, USA), in tapping mode and a Carl Zeiss UltraPlus FESEM (Carl Zeiss AG, Oberkochen, Germany).

Transmission electron microscopy (TEM) was used to characterize the cross section of the thin films. The coatings were performed onto polystyrene coverslips which were cut off and embedded in an epoxy resin. Then, ultrathin cross sections were obtained and immediately mounted onto 200 mesh copper grids. Measurements were performed using transmission electron microscope Carl Zeiss Libra 120 at 80 kV.

## Results and discussion

**Figure 1:**
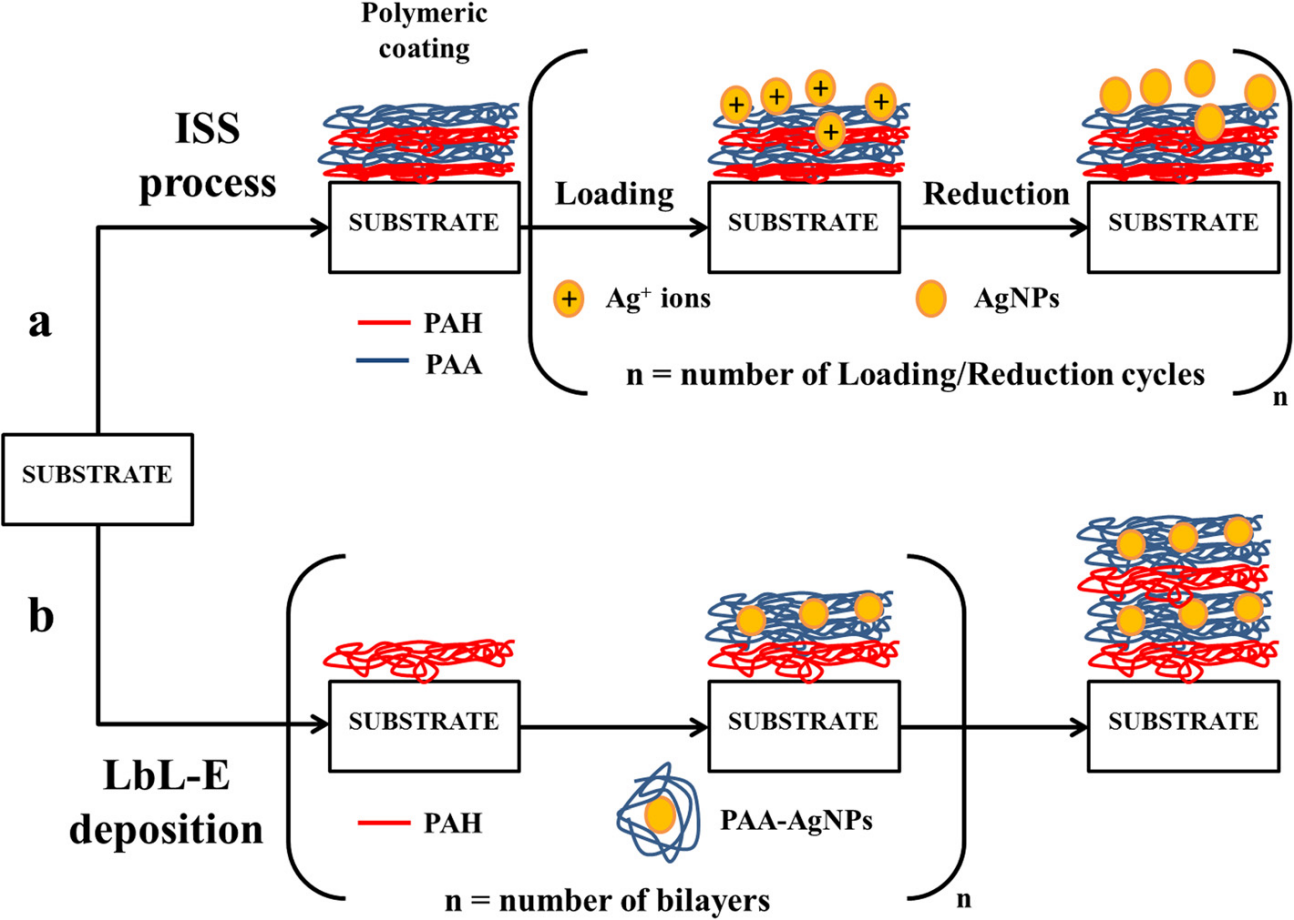
**Schematic representation of the two alternative methods for the synthesis of AgNPs. (a)** ISS process. **(b)** LbL-E deposition technique.

In order to understand the two different chemical synthetic routes (ISS process and LbL-E deposition technique), a schematic representation is shown in Figure 1. In this section, a study of the evolution of the UV-vis absorption bands during the fabrication process, thickness variation, temperature effect, or distribution of the AgNPs into the thin films will be presented. Firstly, the results for the ISS process will be studied and secondly, the results for the LbL-E deposition technique process will be evaluated. Finally, a comparative study about both processes will be shown.

### *In situ* synthesis process of the silver nanoparticles

The weak polyelectrolyte nature of the PAH/PAA matrix makes the pH of the polyelectrolyte dipping solutions determine the number of free carboxylic acid present in the multilayer thin film. The PAA polyanion presents carboxylate and carboxylic acid groups at a suitable pH where the carboxylate groups are responsible for the electrostatic attraction with the cationic groups of the polycation (PAH), forming ion pairs to build sequentially adsorbed multilayers in the LbL assembly. However, the carboxylic acid groups are available for a subsequent ionic exchange for the introduction of inorganic ions such as silver (loading AgNO_3_ solution) and a further *in situ* chemical reduction of the silver cations (Ag^+^) to AgNPs using a reducing agent (reduction DMAB solution). This loading/reduction (L/R) cycles have been repeated up to four times.

**Figure 2:**
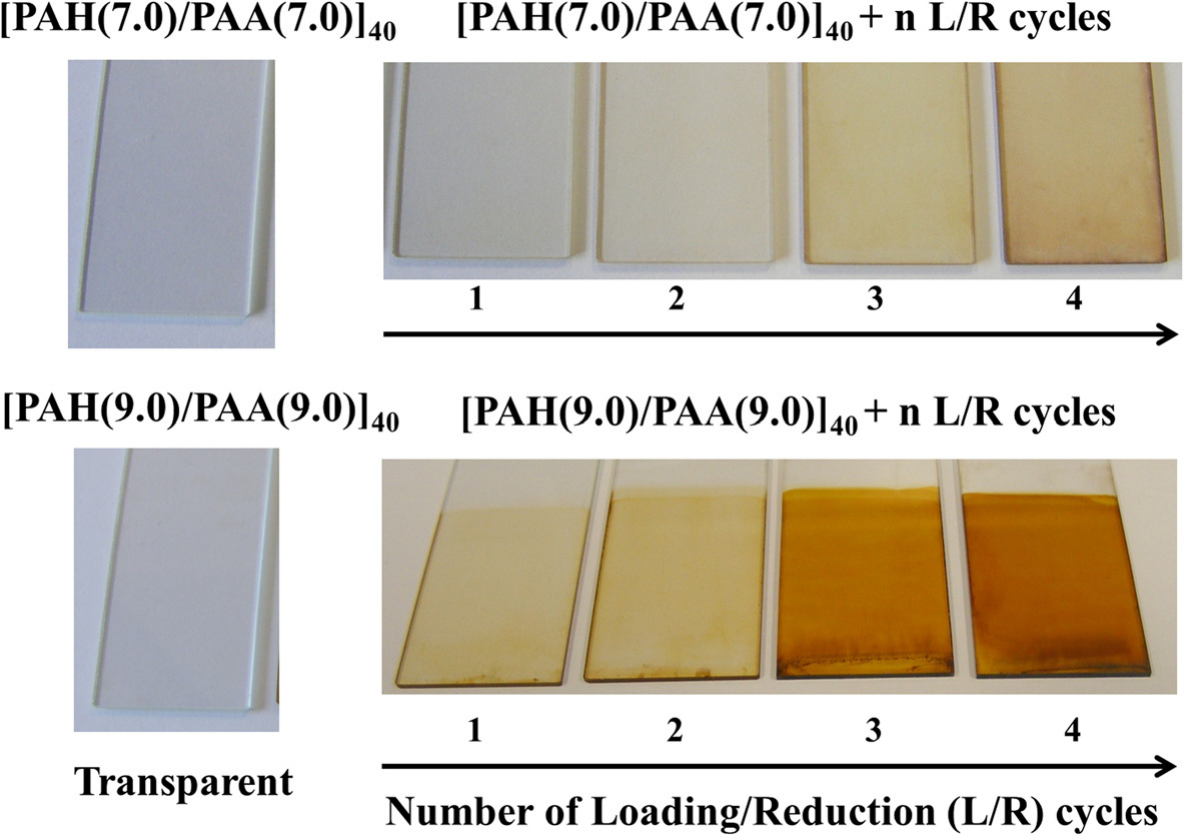
**ISS of the AgNPs into LbL films.** ISS of the AgNPs into LbL films as a function of the number of L/R cycles and the pH (7.0 and 9.0) of the dipping polyelectrolyte solutions (PAH and PAA, respectively).

**Figure 3:**
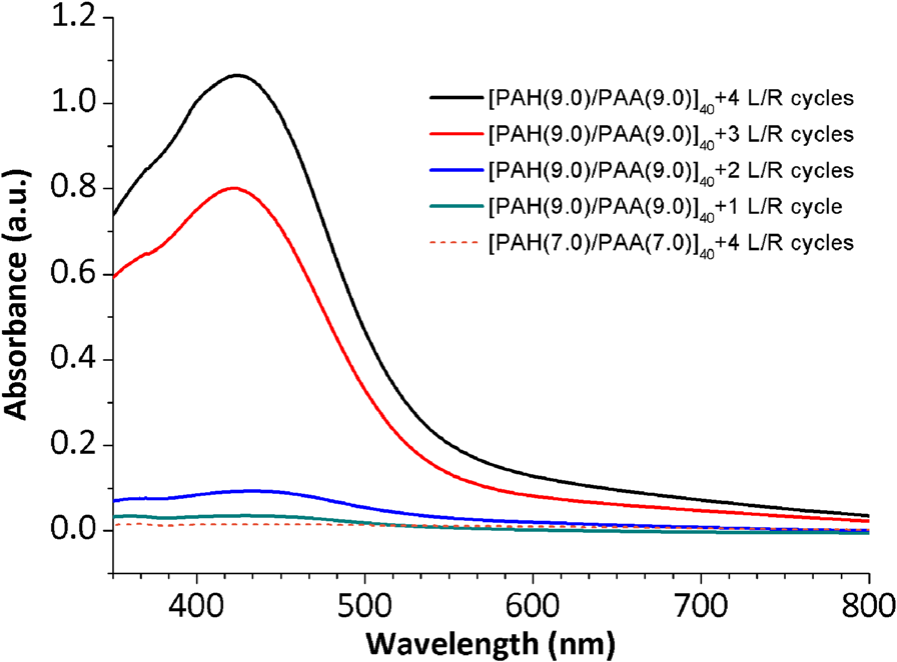
**UV-vis spectra of the ISS process of the AgNPs.** UV-vis spectra of the ISS process of the AgNPs for different number of L/R cycles (1, 2, 3, and 4 L/R) at pH 9.0 (solid lines) and 4 L/R cycles at pH 7.0 (dash line).

In Figure 2, two different pH values of the PAA, pH 7.0 and 9.0, are used to show how the silver nanoparticles are synthesized into the LbL films. A color change from transparent to yellow orange with a characteristic absorption band around 420 nm (see Table 1) has been pointed as an interesting result to corroborate the ISS of the silver nanoparticles into the polymeric film obtained by the LbL assembly. It is possible to appreciate the difference between a glass slide with only polymeric coating [PAH/PAA]_40_ obtained by the LbL assembly at pH 7.0 or 9.0 (totally transparent) and the color evolution after the successive L/R cycles at these two pH values. When a higher number of L/R cycles have been performed, a better definition of the LSPR absorption band around 420 nm can be observed which is indicative that AgNPs have been synthesized in the films. It has been demonstrated that LbL films at pH 9.0 show a dramatically more intense orange coloration in comparison with LbL films at pH 7.0 after the same number of L/R cycles.In Figure 3, UV-vis spectra of the LbL films are shown after the ISS process of the AgNPs from 1 to 4 L/R cycles (solid lines) at pH 9.0 and only for 4 L/R cycles (dash line) at pH 7.0 in order to compare the great difference in intensity of the LSPR absorption band as a function of the pH value.An important consideration is the presence of the LSPR absorption maximum at a wavelength of 424.6 nm which is indicative that AgNPs with a spherical shape have been synthesized into the LbL films. In addition, an increase in the intensity of the LSPR absorption bands at this wavelength position is observed when the number of L/R cycles is increased due to a higher amount of AgNPs incorporated into the LbL films. This aspect was previously corroborated in Figure 2 because the LbL thin films with a higher number of L/R cycles showed a better definition of the orange coloration.

**Table 1:** Thickness evolution of the thin films obtained by ISS process

**Fabrication process**	**Thickness (nm)**	**LSPR (λ**_**max**_**; *A***_**max**_**)**
[PAH(9.0)/PAA(9.0)]_40_	288 ± 5	-
[PAH(9.0)/PAA(9.0)]_40_ + 1 L/R cycle	291 ± 4	421.3 nm; 0.04
[PAH(9.0)/PAA(9.0)]_40_ + 2 L/R cycles	289 ± 16	422.1 nm; 0.09
[PAH(9.0)/PAA(9.0)]_40_ + 3 L/R cycles	296 ± 8	422.8 nm; 0.79
[PAH(9.0)/PAA(9.0)]_40_ + 4 L/R cycles	294 ± 8	424.6 nm; 1.07

Thickness evolution of the ISS films and the location of the LSPR absorption bands (λ_max_) with their maxima absorbance values (*A*_max_).

A study about the thickness evolution of the LbL films before and after the ISS process as well as the maximum wavelength position and absorbance related to the LSPR absorption band is performed, as it can be observed in Table 1. An important consideration is that the resultant thickness after the L/R cycles (from 1 to 4 cycles) is very similar to that of only polymeric LbL coating. As a conclusion, when the number of L/R cycles is increased during the fabrication process, a higher amount of AgNPs are synthesized while the overall thickness of the film remains almost unaltered.

**Figure 4:**
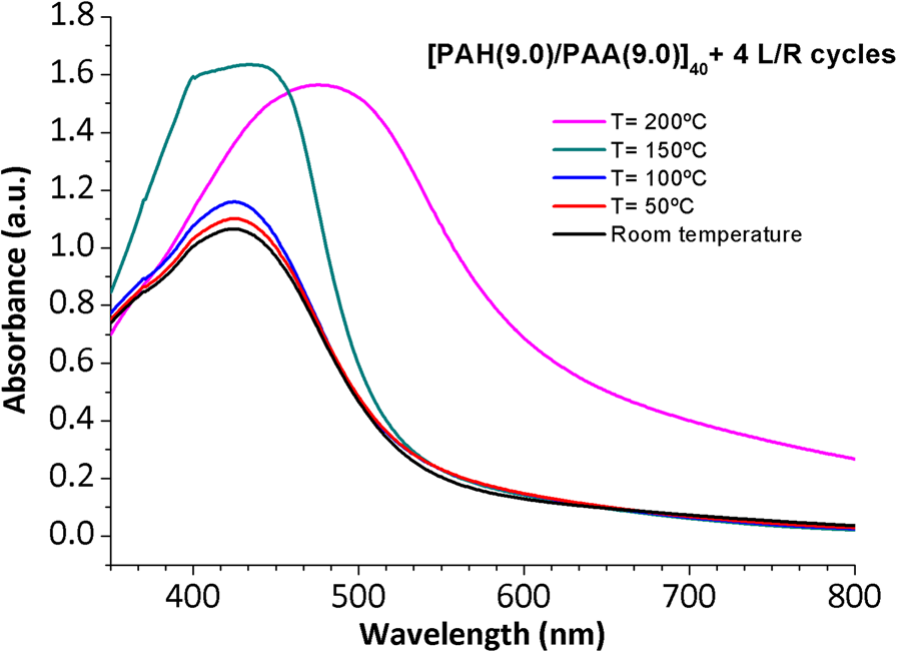
**Evolution of the UV-vis spectra of the thin film [PAH(9.0)/PAA(9.0)]**_**40**_ **+ 4 L/R cycles.** Evolution of the UV-vis spectra of the thin film [PAH(9.0)/PAA(9.0)]_40_ + 4 L/R cycles for a variable range of temperatures from room temperature, 50°C, 100°C, 150°C to 200°C.

As it was previously commented, a thermal post-treatment of the thin films for the higher number of L/R cycles was performed in order to promote a covalent amide bond cross-linking between the polymeric chains of the polyelectrolytes (PAH and PAA), yielding the formation of thin films with a better chemical stability. A variable range of temperature values (50°C, 100°C, 150°C, and 200°C) will be studied and significant differences are observed in the evolution of the LSPR absorption bands, as it can be shown in Figure 4. When the temperature values are varied from room temperature (ambient conditions) to 50°C and 100°C, no changes in the maximal wavelength position of the LSPR absorption bands are observed. For these cases, the LSPR absorption band remains at the same wavelength position (424.6 nm) with a low increase in the maxima absorbance of the LSPR bands when the temperature is increased (50°C and 100°C, respectively). However, a drastic change in the LSPR maximal wavelength position is observed for the higher temperature values where LSPR absorption band is located at 436.8 nm (150°C) and 477.1 nm (200°C) with the corresponding increase in the maxima absorbance values. The films thermally treated at 150°C and 200°C were thinner due to the formation of cross-links via amide bonds between the polyelectrolytes monolayers (PAH and PAA) and as a result, the maxima wavelength position as well as maxima absorbance were increased. In Table 2, a summary of thickness evolution of the thin films as well as the LSPR wavelength positions with their maxima absorbance values are presented as a function of the temperature values.

**Table 2:** Thickness evolution of the thin films obtained by ISS process after thermal treatment

**Fabrication process**	**Temperature**	**Thickness (nm)**	**LSPR (λ**_**max**_**; *A***_**max**_**)**
[PAH(9.0)/PAA(9.0)]_40_+ 4 L/R cycle	Ambient	294 ± 8	424.6 nm; 1.07
[PAH(9.0)/PAA(9.0)]_40_+ 4 L/R cycles	50°C	277 ± 9	424.6 nm; 1.10
[PAH(9.0)/PAA(9.0)]_40_+ 4 L/R cycles	100°C	256 ± 7	424.6 nm; 1.16
[PAH(9.0)/PAA(9.0)]_40_+ 4 L/R cycles	150°C	212 ± 7	436.8 nm; 1.63
[PAH(9.0)/PAA(9.0)]_40_+ 4 L/R cycles	200°C	194 ± 7	477.1 nm; 1.57

Thickness evolution of the ISS thin films and the location of the LSPR absorption bands (λ_max_) with their maxima absorbance values (*A*_max_) as a function of the temperature.

### Layer-by-layer embedding deposition technique

As it was previously commented in the ‘Methods’ section, AgNPs with a specific protective agent (PAA-AgNPs) were firstly synthesized prior to the LbL assembly of the coating [30].

**Figure 5:**
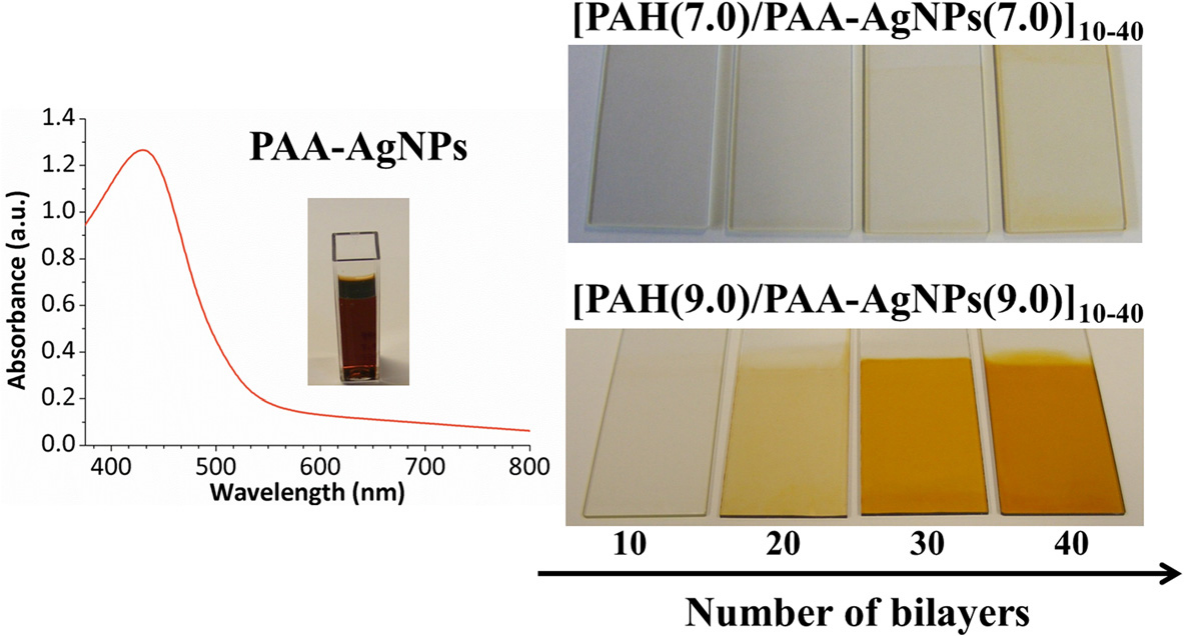
**UV-vis spectroscopy of the PAA-AgNPs and their incorporation into thin films.** UV-vis spectroscopy of the PAA-AgNPs (yellow-orange coloration) and their incorporation into thin films using the LbL-E deposition technique as a function of the pH (7.0 or 9.0) of the dipping polyelectrolyte solutions (PAH and PAA-AgNPs).

Once AgNPs have been synthesized, a further incorporation into thin films is performed using the LbL-E deposition technique [50]. The key of this process is the presence of free anionic carboxylate groups of the PAA at a suitable pH which are the responsible of the electrostatic attraction with cationic polyelectrolytes, such as PAH [41,42]. In this synthetic route, PAA plays a dual role: firstly, preventing the agglomeration of the AgNPs in the LbL film and secondly, making possible to obtain thin films into a desired substrate due to the electrostatic attraction between monolayers of opposite charge [37].In Figure 5, it is possible to appreciate the aspect of the colloidal AgNPs' dispersion (PAA-AgNPs) and their incorporation into thin films using the LbL-E deposition technique as a function of the pH selected (pH 7.0 and 9.0). It is worth noting that UV-vis spectrum corresponding to the PAA-AgNPs shows an intense LSPR absorption band with these coordinates of wavelength position and maximum absorbance (430.6 nm; 1.27). The location of the LSPR absorption band at this specific wavelength position indicates that AgNPs with a spherical shape have been successfully synthesized. In addition, the pH of the PAA-AgNPs is of great interest in order to understand the incorporation of the AgNPs into the films. When the pH is 7.0, the PAA presents less carboxylate groups available and as a result, a lower number of AgNPs have been embedded into the films. However, this aspect drastically changes when the pH of the PAA is higher (pH 9.0) where a higher amount of AgNPs have been incorporated into the LbL-E thin films. A better definition of the orange coloration in the films is observed at pH 9.0 because PAA is building as a fully charged polyelectrolyte and a higher number of carboxylate groups are binding with the cationic polyelectrolyte (PAH) to form ion pairs by electrostatic attraction.

**Figure 6:**
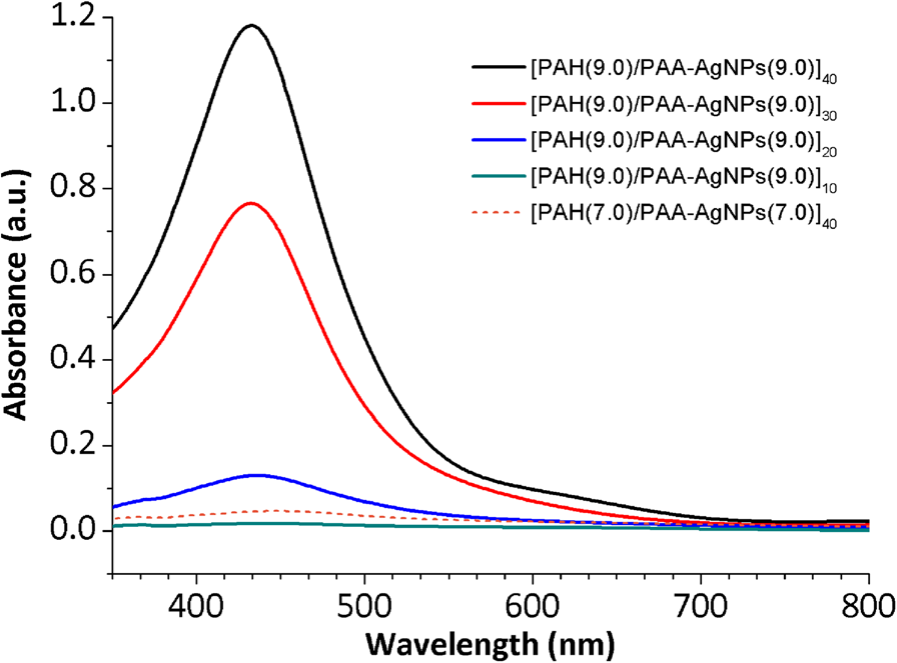
**UV-vis spectra of the LbL-E thin films as a function of the number of bilayers.** UV-vis spectra of the LbL-E thin films as a function of the number of bilayers from 10 to 40 (solid lines) at pH 9.0 and only 40 at pH 7.0 (dash line).

Figure 6 shows the UV-vis spectra of the LbL-E films as a function of the number of bilayers deposited (10, 20, 30, and 40) at pH 9.0 (solid lines) and only 40 at pH 7.0 (dash line). A better definition in the intensity of the LSPR absorption band around 430 nm is observed when a higher number of bilayers are deposited from 10 to 40, which it is indicative that a higher number of AgNPs are incorporated. In addition, the LSPR of the AgNPs into the LbL-E films appears at the same wavelength position to that PAA-AgNPs and as a conclusion, no aggregation of AgNPs is observed in the LbL films due to PAA acting as a protective agent and preventing the agglomeration of the AgNPs during the fabrication process. A study about the thickness evolution of the LbL-E films during the fabrication is performed (see Table 3). As it was expected, an increase of the resultant thickness is observed when the number of bilayers is increased for 10 to 40.

**Table 3:** Thickness evolution of the thin films obtained LbL-E deposition technique

**Fabrication process**	**Thickness (nm)**	**LSPR (λ**_**max**_**; *A***_**max**_**)**
[PAH(9.0)/PAA-AgNPs(9.0)]_10_	63 ± 5	421.3 nm; 0.017
[PAH(9.0)/PAA-AgNPs(9.0)]_20_	165 ± 4	432.1 nm; 0.13
[PAH(9.0)/PAA-AgNPs(9.0)]_30_	507 ± 16	432.3 nm; 0.77
[PAH(9.0)/PAA-AgNPs(9.0)]_40_	642 ± 12	432.6 nm; 1.18

Thickness evolution of the LbL-E thin films and the location of the LSPR absorption bands (λ_max_) with their maxima absorbance values (*A*_max_).

**Figure 7:**
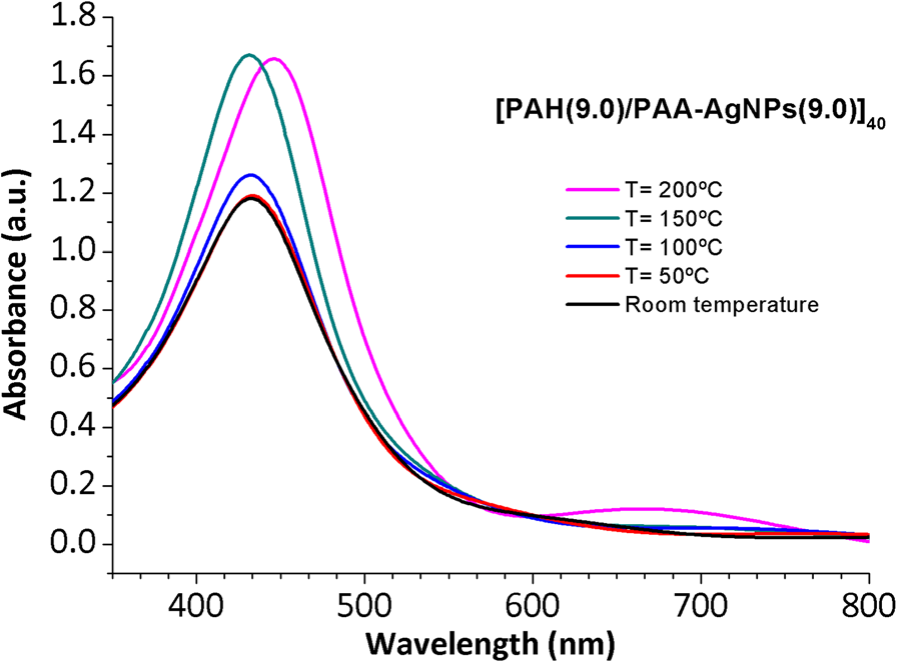
**Evolution of the UV-vis spectra of the thin film [PAH(9.0)/PAA-AgNPs(9.0)]**_**40**_**.** Evolution of the UV-vis spectra of the thin film [PAH(9.0)/PAA-AgNPs(9.0)]_40_ for a variable range of temperatures from room temperature, 50°C, 100°C, 150°C, to 200°C.

The influence of the temperature in the LbL-E thin films has been studied. As it was previously performed in the ISS process, the LbL-E films were thermally treated at the same variable temperature values from 50°C to 200°C in order to promote an amide bond cross-link of the polymeric chains. In Figure 7, it is possible to appreciate the evolution of the LSPR absorption band which it remains at the same wavelength position (432.6 nm) from room temperature to the thermal treatment at 150°C. However, a shift in the wavelength position of the LSPR absorption band is observed from 432.6 to 446.9 nm for the higher temperature value (200°C) by forming amide bonds (cross-linked films) with the corresponding partial reduction thickness in comparison with untreated films. In addition, in all the cases of the study, an increase in the maxima absorbance of the LSPR absorption bands is observed after thermal treatment. In Table 4, a summary of thickness evolution of the LbL-E thin films as well as the LSPR wavelength positions with their maxima absorbance values is presented as a function of the temperature.

**Table 4:** Thickness evolution of the thin films obtained LbL-E deposition technique after thermal treatment

**Fabrication process**	**Temperature**	**Thickness (nm)**	**LSPR (λ**_**max**_**; *A***_**max**_**)**
[PAH(9.0)/PAA-AgNPs(9.0)]_40_	Ambient	642 ± 12	432.6 nm; 1.18
[PAH(9.0)/PAA-AgNPs(9.0)]_40_	50°C	611 ± 16	432.6 nm; 1.20
[PAH(9.0)/PAA-AgNPs(9.0)]_40_	100°C	600 ± 12	432.6 nm; 1.26
[PAH(9.0)/PAA-AgNPs(9.0)]_40_	150°C	552 ± 9	432.6 nm; 1.68
[PAH(9.0)/PAA-AgNPs(9.0)]_40_	200°C	452 ± 10	446.9 nm; 1.66

Thickness evolution of the LbL-E thin films and the location of the LSPR absorption bands (λ_max_) with their maxima absorbance values (*A*_max_) as a function of the temperature.

### A comparative study between ISS process and LbL-E deposition technique

In this section, a comparative study about both processes will be shown for a better understanding of the incorporation of AgNPs into thin films using wet chemistry reactions. In order to establish any significant differences, the evolution of the thin films will be studied for the higher number of bilayers and L/R cycles at room temperature (ambient) and after thermal post-treatment of 200°C. In addition, a study about the distribution of the AgNPs into the thin films will be necessary to understand the shift of the LSPR absorption bands.

**Figure 8:**
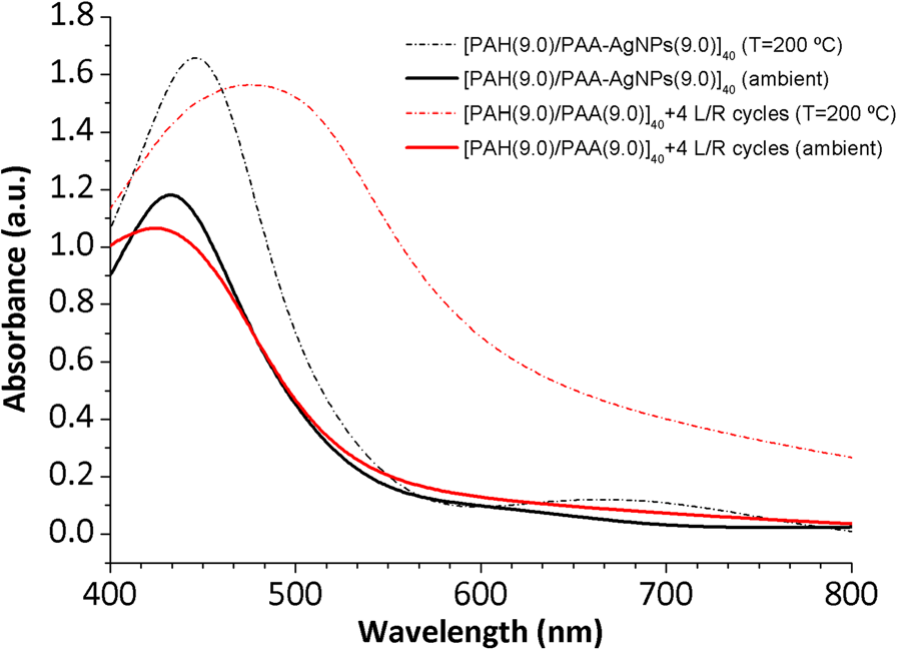
**Evolution of the UV-vis spectra of thin films obtained by ISS and LbL-E deposition technique.** Evolution of the UV-vis spectra of the thin films obtained by ISS process and LbL-E deposition technique as a function of two temperatures values (ambient and 200°C).

**Figure 9:**
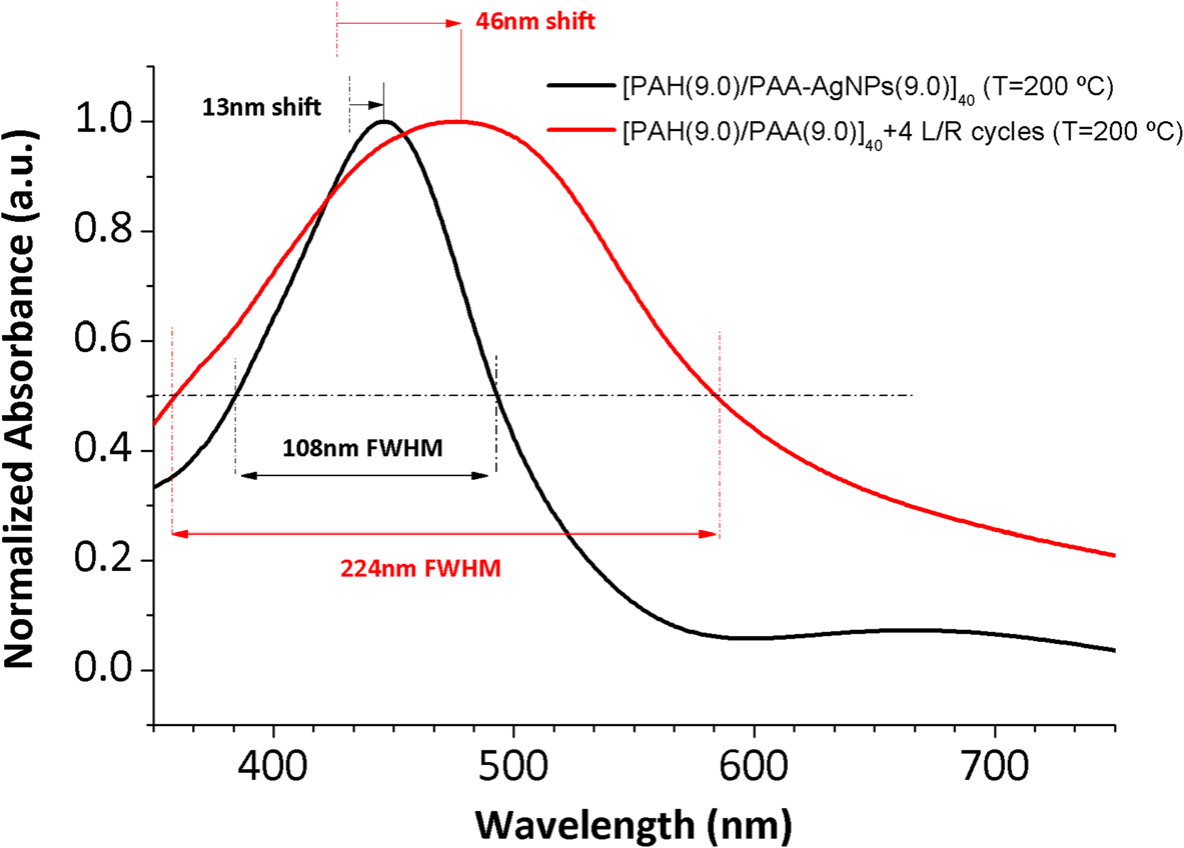
**Normalized UV-vis spectra for ISS and LbL-E films after thermal post-treatment.** Normalized UV-vis spectra for ISS and LbL-E films after thermal post-treatment (200°C) with their maximal wavelength shift and their FWHM.

**Figure 10:**
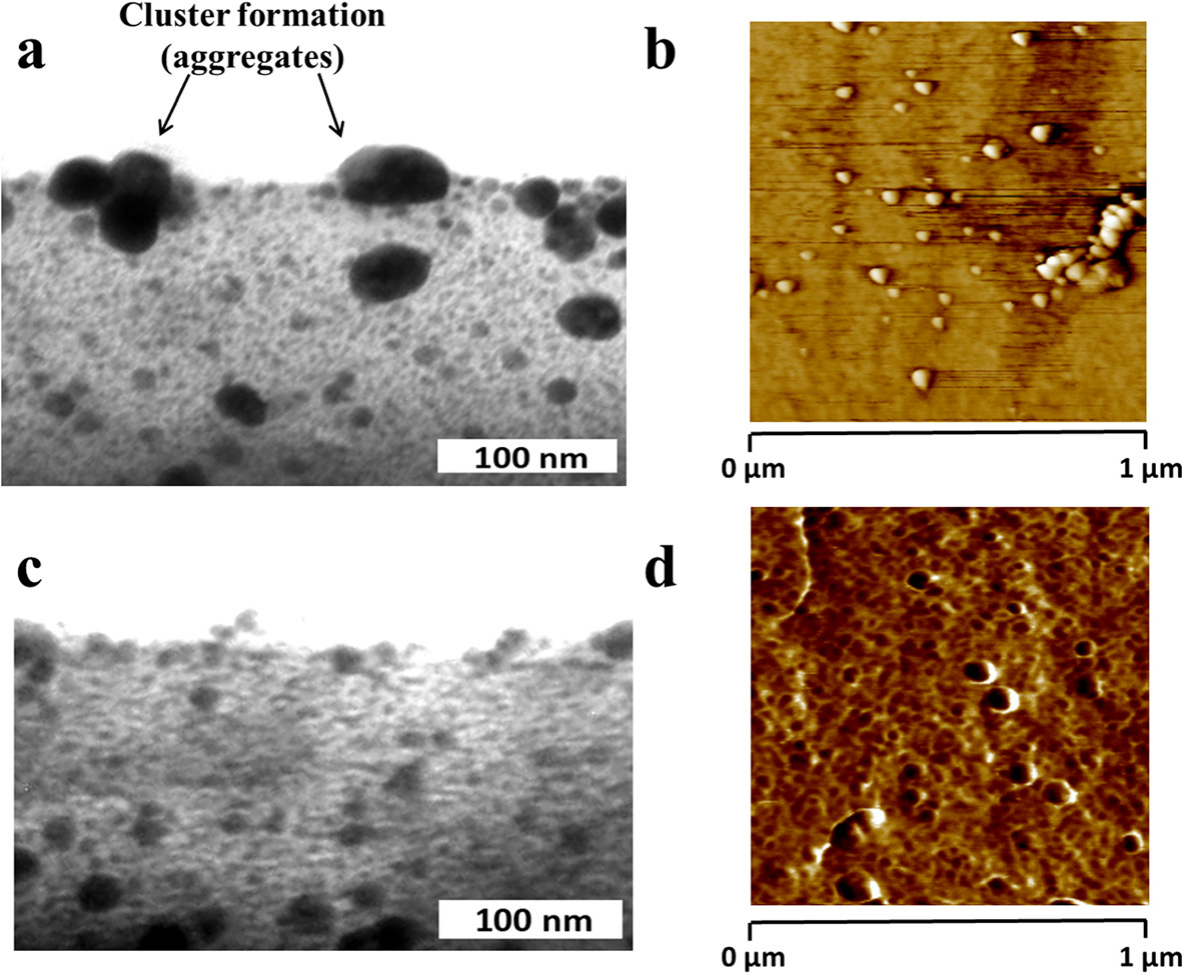
**Cross-sectional TEM micrographs of the upper part of the thin film and AFM phase images. (a, b)** Cross-sectional TEM micrograph of the upper part of the thin film and AFM surface phase image for the ISS process. **(c, d)** Cross-sectional TEM micrograph of the upper part of the thin film and AFM surface phase image for the LbL-E deposition technique.

**Figure 11:**
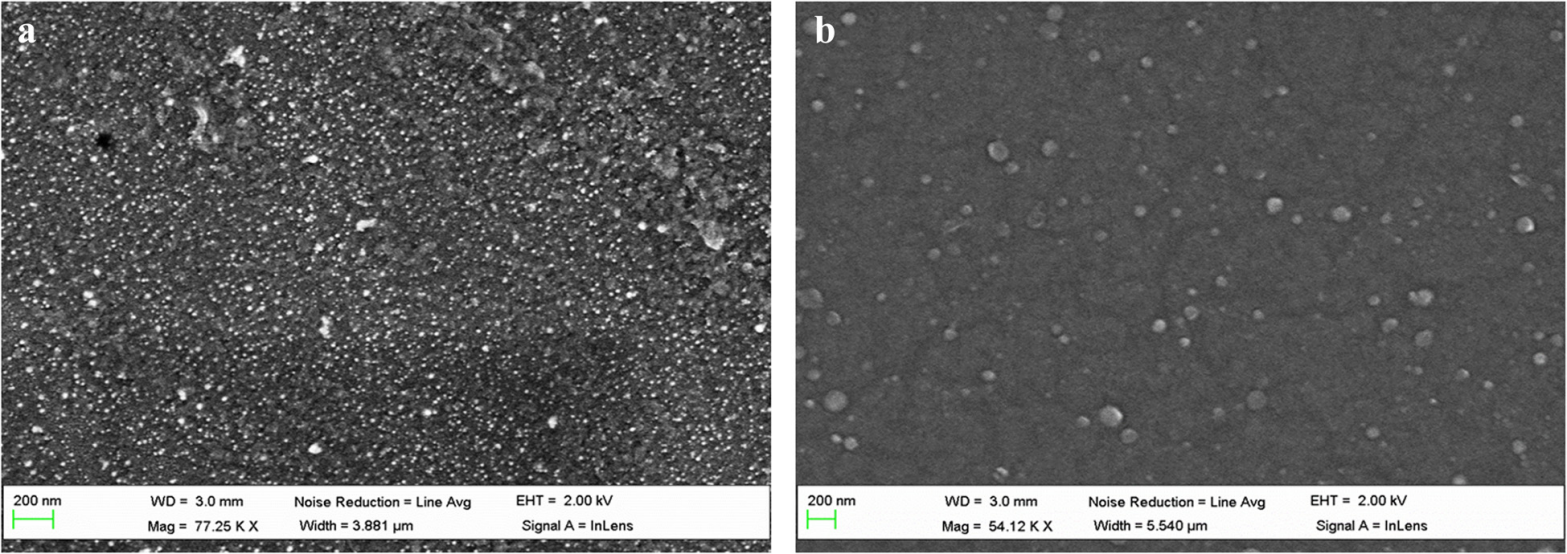
**SEM images of the thin films. (a)** ISS process. **(b)** LbL-E deposition technique.

Figure 8 shows the UV-vis spectra of the thin films obtained by ISS process and LbL-E deposition technique before and after thermal post-treatment (200°C). First of all, the location of the LSPR absorption band without thermal treatment for the ISS process appears at a shorter wavelength position (424.6 nm) in comparison with the LbL-E deposition technique (432.6 nm). This aspect related to the wavelength location of the LSPR absorption band shows a high dependence with the size of the AgNPs in the films. When AgNPs of higher size are incorporated into thin films, LSPR absorption band is located at higher wavelength position as it occurs in the LbL-E deposition technique. However, when smaller AgNPs are incorporated into the films, the LSPR absorption band is located at a lower wavelength position as it occurs in the ISS process. In addition, a shift of the LSPR absorption bands is observed in both processes after thermal post-treatment, being more notorious for the ISS process. One of the reasons of this displacement in wavelength is the better proximity of the AgNPs because of the partial thickness reduction after thermal post-treatment (confirmed in Tables 2 and 4) and as a result, the maxima absorbance values of the LSPR bands are increased.In Figure 9, normalized UV-vis spectra for the ISS and LbL-E films are shown after thermal post-treatment where it is possible to appreciate their maximal wavelength shifts respect untreated films (ambient) and the full width at half maximum (FWHM). The maximal wavelength shift is only 13 nm for the LbL-E films, whereas the shift for the ISS process is 46 nm. This great difference between both processes is associated to the use of a specific protective agent (PAA-AgNPs) in the LbL-E films, which prevents the agglomeration of the AgNPs during the fabrication process and after thermal post-treatment. However, ISS process shows a higher maximal wavelength shift because AgNPs are randomly synthesized into the polymeric matrix without any control in their distribution and aggregation state. This aspect related to the aggregation of the AgNPs into the films is corroborated by FWHM which it is duplicated for the ISS process (224 nm) in comparison with the LbL-E deposition technique (108 nm). In addition, the widening of the LSPR absorption band for the ISS is associated to the presence of AgNPs with a variable size (polydispersity) or to the presence of silver clusters (aggregates) in the films. However, LbL-E films show the possibility of incorporating AgNPs with a desired size (monodispersity) and perfectly encapsulated PAA-AgNPs and due to this, no aggregation of the AgNPs is observed after thermal post-treatment.In order to corroborate this hypothesis related to the size, aggregation, and distribution of the AgNPs into the thin films, cross-sectional TEM micrographs of the upper part of the thin film close to the surface as well as AFM phase images (1 × 1 μm) in tapping mode for the ISS and LbL-E films were taken, as it can be observed in Figure 10. The cluster formation is perfectly observed in the cross-sectional TEM micrograph (Figure 10a) for the ISS process, mostly in the outer surface of the film. In addition, AFM phase image (Figure 10b) reveals the presence of AgNPs with variable size and random distribution which are mixed with clusters in the specific zones of the topographic distribution. This aggregation in the film has a significant influence in the maximal wavelength position of the LSPR absorption band, corroborated by UV-vis spectra. Finally, the cross-sectional TEM image (Figure 10c) for the LbL-E film shows a gradual incorporation of AgNPs from the inner to the outer surface of the film, and AFM phase image in Figure 10d reveals that no aggregation of AgNPs is observed in the topographic distribution. An important consideration is that the size of the AgNPs using LbL-E is higher than the size observed in the ISS process, whereas a high amount of AgNPs are synthesized using the ISS process.This aspect related to the amount and size of the AgNPs is corroborated by SEM images. In Figure 11a, it is possible to appreciate that a higher amount of smaller AgNPs size is obtained for the ISS process. In opposition to this, the LbL-E deposition technique (Figure 11b) shows the incorporation of AgNPs with a higher size in the topographic distribution of the films.

As a conclusion of both processes, the use of PAA as a protective agent of the AgNPs in the LbL-E deposition technique is of vital importance because it can prevent cluster formation along the coating, although it is possible to appreciate nanoparticles of higher size along the coating thickness. To sum up and according to the results, LbL-E deposition technique allows the incorporation of AgNPs of higher size along the film, whereas cluster formation mixed with AgNPs of small size is only observed for the ISS process.

## Conclusions

This work is based on the synthesis and incorporation of silver nanoparticles into thin films using two alternative techniques with remarkable differences, the ISS process and the LbL-E deposition technique. Firstly, both processes are separately analyzed as a function of several parameters such as the pH value of the dipping polyelectrolyte solutions, thickness evolution, or temperature effect. Secondly, a comparative study between both processes has been performed in order to establish the difference in the size and distribution of the nanoparticles into the LbL films.

In both methodologies, the presence of a weak polyelectrolyte such as poly(acrylic acid, sodium salt) is the key for synthesizing metallic silver nanoparticles due to its pH-dependent behavior, making possible to obtain carboxylate and carboxylic acid groups as a function of the pH value. For the ISS process, the presence of free carboxylic acid groups is the key for the introduction of silver ions which are further reduced to silver nanoparticles. However, in the case of the LbL-E deposition technique, PAA is acting as an encapsulating agent of the nanoparticles and these AgNPs are incorporated into thin films by the electrostatic attraction between the polycation (PAH), and the carboxylate groups of the PAA capped the nanoparticles (PAA-AgNPs).

The location of the LSPR absorption bands varies from 424.6 nm for the ISS process to 432.6 nm for the LbL-E deposition technique. However, a post-thermal treatment produces a wavelength shift of the LSPR absorption bands, being more significant for the ISS process because the LSPR maximum wavelength position is displaced at 46 nm in comparison with only 13 nm in the LbL-E deposition technique. In addition, the full width at half maximum is higher for the ISS film (224 nm) in comparison with the LbL-E film (108 nm).

A morphological characterization (SEM, TEM, or AFM) is performed in order to clarify the size and distribution of the nanoparticles in the LbL films. SEM images indicate that a higher amount of AgNPs with less size is synthesized for the ISS process. Cross-sectional TEM micrographs and AFM phase images indicate the cluster formation of AgNPs in the topographic distribution of the ISS process which is not observed in the LbL-E films. These remarkable differences between both processes related to the distribution, size, and partial aggregation have a considerable influence in the final location of the LSPR absorption bands. In addition, the great importance of using a protective agent such as PAA-AgNPs in the LbL-E deposition technique is to prevent the aggregation of the AgNPs during the fabrication process and after thermal post-treatment. To our knowledge, this is the first time that a comparative study of the synthesis and incorporation of AgNPs into thin films is presented in the bibliography using two alternative methods with the same chemical reagents based on wet chemistry.

## Competing interests

The authors declare that they have no competing interests.

## Authors’ contributions

PJR carried out the main part of the experimental work. He participated in the design of the study and in the draft of the manuscript. JG participated in the experimental work, carried out the AFM images and contributed with the draft of the manuscript. IRM participated in the design of the study. FJA participated in the design of the study and helped to draft the manuscript. All authors read and approved the final manuscript.
